# ZD7288 Enhances Long-Term Depression at Early Postnatal Medial Perforant Path-Granule Cell Synapses

**DOI:** 10.1155/2012/237913

**Published:** 2012-06-26

**Authors:** Xiati Guli, Tursonjan Tokay, Marco Rohde, Roland A. Bender, Rüdiger Köhling, Timo Kirschstein

**Affiliations:** ^1^Oscar Langendorff Institute of Physiology, University of Rostock, Gertrudenstraße 9, 18057 Rostock, Germany; ^2^Institute of Neuroanatomy, University of Hamburg, Martinistraße 52, 20246 Hamburg, Germany

## Abstract

Hyperpolarization-activated, cyclic nucleotide-gated nonselective (HCN) channels modulate both membrane potential and resistance and play a significant role in synaptic plasticity. We compared the influence of HCN channels on long-term depression (LTD) at the medial perforant path-granule cell synapse in early postnatal (P9–15) and adult (P30–60) rats. LTD was elicited in P9–15 slices using low-frequency stimulation (LFS, 900 pulses, 1 Hz; 80 ± 4% of baseline). Application of the specific HCN channel blocker ZD7288 (10 **μ**M) before LFS significantly enhanced LTD (62 ± 4%; *P* < 0.01), showing HCN channels restrain LTD induction. However, when ZD7288 was applied after LFS, LTD was similar to control values and significantly different from the values obtained with ZD7288 application before LFS (81 ± 5%; *P* < 0.01), indicating that HCN channels do not modulate LTD expression. LTD in slices from adult rats were only marginally lower compared to those in P9–15 slices (85 ± 6%), but bath application of ZD7288 prior to LFS resulted in the same amount of LTD (85 ± 5%). HCN channels in adult tissue hence lose their modulatory effect. In conclusion, we found that HCN channels at the medial perforant path-granule cell synapse compromise LFS-associated induction, but not expression of LTD in early postnatal, but not in adult, rats.

## 1. Introduction

Hyperpolarization-activated, cyclic nucleotide-gated nonselective (HCN) channels show a considerable conductance at the resting membrane potential and mediate a slowly inactivating and inwardly rectifying Na^+^ current [1, 2]. Hence, these channels appear to be ideal modulators of both the membrane potential and the membrane resistance of neurons and thus may play a significant role in synaptic transmission. In recent years, it has become evident that HCN channels are also relevant modulators of synaptic plasticity [3–5]. HCN channels may have differential effects on different synapses and/or various types of synaptic plasticity, depending on, for example, the synapse type, pre- or postsynaptic expression patterns, and ontogenetic stage. Thus, while long-term potentiation (LTP) following electrical stimulation to the Schaffer collateral input of CA1 pyramidal cells was shown to be unaltered in HCN1-deficient adult mice, a significant enhancement of LTP was observed when the perforant path input to CA1 was stimulated [3]. Such a constraining role of HCN channels on synaptic plasticity in CA1 was also found with long-term depression (LTD) following pharmacological activation of group I metabotropic glutamate receptors [5]. However, LTD following electrical stimulation at the same synapse was also assessed in the same study and failed to be affected by HCN channel inhibition. Since the expression pattern of HCN channels changes during development—presynaptic HCN channels are expressed on glutamatergic axon terminals of the medial perforant path (MPP) input to dentate gyrus granule cells only during the first 2-3 weeks of postnatal development and subsequently are lost during maturation [4]—comparing plasticity in immature and mature tissue allows to dissect the impact of pre- and postsynaptic HCN channel expression. In immature tissue, HCN channel-mediated effects will be predominantly presynaptically driven, in adult tissue, postsynaptically. Interestingly, short-term depression (STD) at the immature synapse was enhanced following pharmacological HCN channel inhibition [4], as a first indication of a constraining role of HCN channels on synaptic plasticity. In the present study, we assessed NMDA receptor-dependent low-frequency stimulation- (LFS-) induced LTD at the MPP-granule cell synapse at different developmental stages and found an enhancement of LTD following HCN channel inhibition in tissue from early postnatal, but not in tissue from adult animals. Moreover, this effect was mimicked by nitric oxide synthase inhibition. These findings emphasize the modulating role of HCN channels on synaptic plasticity and provide evidence for an important role of presynaptic HCN channels in activity-dependent changes of synaptic transmission. They also suggest that nitric oxide may play a role as a retrograde messenger that links postsynaptic NMDA-receptor activation to presynaptic HCN channel function.

## 2. Materials and Methods

### 2.1. Animals and Slice Preparation

All experiments were performed with either early postnatal (P9–15) or adult (P30–60) male CD rats purchased from Charles River (Sulzfeld, Germany) and conformed to national and international guidelines on the ethical use of animals (European Council Directive 86/609/EEC). After deep anesthesia with diethyl ether (Mallinckrodt Baker, Deventer, The Netherlands), rats were decapitated and the brain was rapidly removed and submerged into oxygenated ice-cold dissection solution containing (in mM) 125 NaCl, 26 NaHCO_3_, 3 KCl, 1.25 NaH_2_PO_4_, 0.2 CaCl_2_, 5 MgCl_2_, and 13 D-glucose (gassed with 95% O_2_, 5% CO_2_; pH 7.4). Subsequently, horizontal brain slices (400 *μ*m) of the hippocampus were prepared in this ice-cold dissection solution with a vibratome (Integraslice 7550MM, Campden Instruments, Loughborough, UK). The hippocampal formations of both hemispheres were carefully excised and then transferred into a holding chamber at room temperature filled with artificial cerebrospinal fluid (ACSF) containing (in mM) 125 NaCl, 26 NaHCO_3_, 3 KCl, 1.25 NaH_2_PO_4_, 2.5 CaCl_2_, 1.3 MgCl_2_, and 13 D-glucose (gassed with 95% O_2_, 5% CO_2_; pH 7.4).

### 2.2. Electrophysiological Recording and LTD Induction

For electrophysiological recordings, hippocampal slices were transferred into an interface chamber (BSC-HT, Harvard Apparatus, Holliston, USA) maintained at 32°C (TC-10, npi electronic GmbH, Tamm, Germany) and superfused with ACSF (composition as above, perfusion rate of 2-3 mL/min). Field excitatory postsynaptic potentials (fEPSPs) were recorded using borosilicate glass pipettes (GB150-8P, Science Products, Hofheim am Taunus, Germany) with a tip resistance of 2-3 MΩ (fabricated with PIP5 puller from HEKA Elektronik, Lambrecht, Germany) and filled with ACSF. Bipolar stimulating electrodes were made from Teflon-insulated platinum wires (PT-2T, Science Products), and double-pulse stimulation (interstimulus interval 40 ms) was delivered using a Master-8 stimulator (A.M.P.I., Jerusalem, Israel) and a stimulus isolator (A365, World Precision Instruments, Sarasota, USA) at a baseline rate of 0.033 Hz. The baseline stimulation strength was adjusted to 30–40% of the maximal fEPSP amplitude and typically in the range of 50–100 *μ*A. The evoked potentials were amplified and filtered at 1 kHz (EXT-10-2F, npi), digitized (Micro1401, CED, Cambridge Electronic Design, Cambridge, UK) and stored for off-line analysis (Signal 2.16, CED). 

To record from the medial perforant path-granule cell synapse, both the stimulating and the recording electrodes were placed into the middle molecular layer (MML) of the dentate gyrus. Since these synapses typically display paired-pulse depression [6, 7], double-pulse stimulation (interstimulus interval of 40 ms) was used during the initial 5–7 minutes of each experiment, and the paired-pulse ratio (PPR) was calculated as the ratio of the 2nd pulse-evoked fEPSP to the 1st pulse-evoked fEPSP. In addition, gabazine (1 *μ*M, Tocris, Bristol, UK) was added to the ACSF in order to reduce contaminations by stimulation of local GABAergic interneuronal circuits, and single stimulation was used for the remaining experiment. Short-term depression (STD) and long-term depression (LTD) were induced by a low-frequency stimulation (LFS) protocol consisting of 900 pulses at 1 Hz (baseline stimulation strength, 1.5-fold pulse width). STD (average of fEPSP amplitudes at 0–2 min after LFS) and LTD (average of fEPSP amplitudes at 55–60 minutes after LFS) values are given as fEPSP amplitudes expressed as the percentage of the baseline response. The influence of HCN channels on LFS-induced LTD was tested by bath application of ZD7288 (Tocris) which was freshly dissolved in ACSF (10 *μ*M). The specific N-methyl-D-aspartate (NMDA) receptor blocker D-2-amino-5-phosphonopentanoate (D-AP5) and the nitric oxide synthase (NOS) blocker N^G^-nitro-L-arginine methyl ester (L-NAME) were also obtained from Tocris. All other chemicals were purchased from Sigma (Taufkirchen, Germany). Since ZD7288 was shown to depress glutamatergic receptors [8, 9], we performed interleaved experiments without LFS that were used to normalize the STD and LTD values. This normalization was performed by calculating the ratio of the LFS-treated by the non-LFS-treated control value and expressed as the percentage of baseline response.

### 2.3. Statistical Analysis

All data are expressed as means ± SEM. Statistical comparisons were performed using Student's two-tailed *t*-test. Significant differences are indicated by asterisks (*P < 0.05; **P < 0.01; ***P < 0.001) for comparisons between untreated and treated slices (unpaired test) and by diamonds (^*◊*^ *P* < 0.05; ^*◊◊*^ *P* < 0.01) for comparisons between different time points within the same group (paired test).

## 3. Results

### 3.1. LFS-Induced LTD in Early Postnatal Rats Is Enhanced by ZD7288

**Figure 1:**
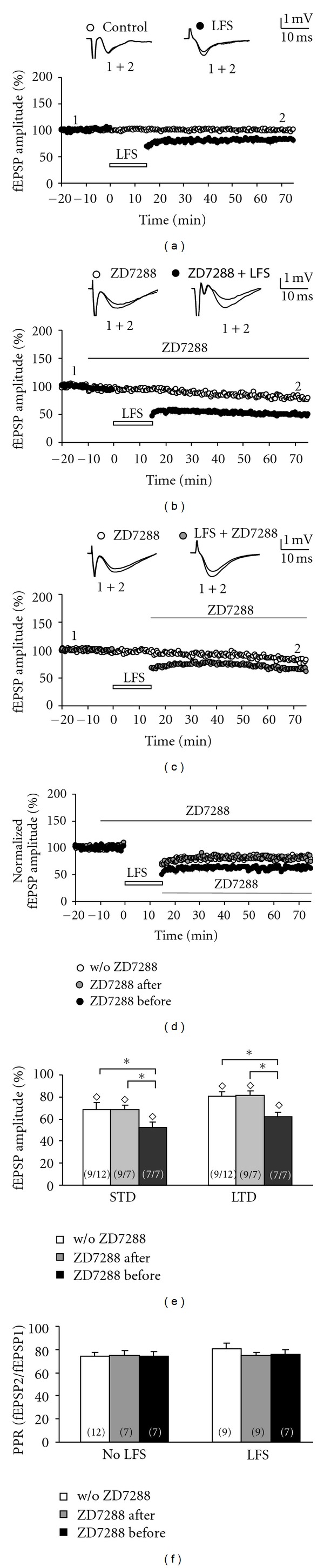
ZD7288 enhances LFS-induced LTD in early postnatal rats. (a) The time course of the excitatory postsynaptic field potential (fEPSP) amplitude following stimulation of the medial perforant path (MPP) in early postnatal rats (P9–15) displays LTD (closed symbols) following low-frequency stimulation (LFS, indicated by an open bar). Time-control experiments without LFS (open symbols) confirmed stable recording conditions. Representative fEPSPs were taken at the beginning (indicated by “1”) and at the end (indicated by “2”) of the experiment. (b) The time course of the fEPSP amplitude following ZD7288 treatment (starting 10 min prior to LFS, indicated by a closed bar) shows an enhanced depression in LFS-treated slices (closed symbols). Non-LFS-treated time-control experiments (open symbols) showed a synaptic depression after ZD7288 application. Note the large difference of fEPSP amplitudes at the end of the experiment which was more pronounced than that without ZD7288 application in (a). (c) Comparing the fEPSP amplitude time course following ZD7288 treatment (starting after LFS, indicated by a gray bar) between LFS-treated (gray symbols) and non-LFS-treated time-control experiments (open symbols) reveals a synaptic depression which was similar to that in non-ZD7288 conditions shown in (a). (d) Normalized time courses of fEPSP amplitudes in three different conditions: without ZD7288 (open symbols), ZD7288 before LFS (closed symbols), and ZD7288 after LFS (gray symbols). Note that similar levels of LFS-induced STD and LTD were obtained in both slices without ZD7288 and slices with ZD7288 after LFS. (e) The bar graph summarizes the STD and LTD levels of three different conditions. The number of slices is given in parentheses (number of LFS-treated slices/number of non-LFS-treated slices). (f) The bar graph summarizes the paired-pulse ratio of all experimental groups recorded at the beginning of each experiment, that is, before ZD7288 and/or LFS were applied to the slices. Note that the PPR was similar in all groups. All data are mean ± SEM.

Long-term depression (LTD) in the hippocampus is commonly induced by low-frequency stimulation (LFS). Here, we employed an LFS protocol of 900 pulses at 1 Hz (indicated by an open bar in Figure 1(a)). Such a paradigm applied to the medial perforant path (MPP) input onto dentate gyrus-granule cells caused an immediate synaptic depression to 70 ± 6% of the baseline fEPSP amplitude (Figure 1(a), closed symbols, *n* = 9). During the followup of the experiment, this short-term depression (STD) resulted in stable LTD of 80 ± 4% of baseline at 60 minutes after LFS. Interleaved time-control experiments without LFS demonstrated that our recording conditions were stable for at least 90 minutes (100 ± 1% at the end of the experiment; open symbols in Figure 1(a), *n* = 12). Since we were interested in the effect of 10 *μ*M ZD7288 on LFS-induction of LTD, this compound was bath applied 10 minutes prior to LFS (Figure 1(b), closed symbols, *n* = 7). In these experiments, fEPSP amplitudes both immediately after LFS (STD) and at the end of the experiment (LTD) were more markedly depressed (51 ± 5% and 48 ± 3%, *n* = 7). However, this enhanced STD and LTD could be biased by the synaptic depression following ZD7288 [8, 9]. Therefore, we performed again interleaved time-control experiments without LFS (Figure 1(b), open symbols, *n* = 7) and indeed obtained a synaptic depression when ZD7288 was bath applied to the slices (80 ± 2% at the end of the experiment; open symbols in Figure 1(b)). In order to directly compare LFS-induced LTD with and without HCN channel inhibition, we normalized LFS-treated slices (closed symbols in Figures 1(a) and 1(b)) to non-LFS-treated slices (open symbols in Figures 1(a) and 1(b)). This normalization indeed revealed that both STD and LTD were significantly enhanced by ZD7288 preapplication as compared to interleaved time-control experiments (STD with ZD7288: 53 ± 5%, STD control: 69 ± 6%; *P* < 0.05; LTD with ZD7288: 62 ± 4%, LTD control: 80 ± 4%, *P* < 0.01; Figures 1(d) and 1(e)). These results so far indicate that HCN channel activation during LFS appears to constrain both STD and LTD.

We then asked the question whether ZD7288 would also exert this effect when applied after LFS has been completed. This is an important control experiment since STD—in this case—is recorded before ZD7288 could actually depress synaptic transmission and thus should not be altered by HCN channel inhibition (Figure 1(c)). In fact, STD was unaltered by ZD7288 treatment after LFS (68 ± 5%, *n* = 9, gray symbols in Figure 1(c)). But importantly, fEPSP amplitudes at the end of the experiment were 65 ± 4% (*n* = 9) in LFS-treated slices and 81 ± 3% in non-LFS-treated control experiments (open symbols in Figure 1(c)). Following normalization, both STD and LTD were no longer significantly different from control experiments without ZD7288 application (STD: 68 ± 5%, *P* < 0.05 versus preapplied ZD7288; LTD: 81 ± 5%, *P* < 0.01 versus preapplied ZD7288; Figures 1(d) and 1(e)). On the one hand, this experiment suggests that the LTD enhancement is a specific ZD7288-mediated effect and not an artifact due to normalization procedure. On the other hand, these data also indicate that HCN channel activation modulate the induction but not the expression of LFS-induced LTD.

Another important pitfall in recording MPP-evoked fEPSPs is the potential contamination by field potentials following stimulation of the adjacent fiber tract—the lateral perforant path (LPP). The propensity of LTD to be obtained in the middle molecular layer may actually vary with the degree of contamination by LPP stimulation. Therefore, we analyzed the paired-pulse ratio (PPR) in all six experimental groups at the beginning of the experiment, that is, before LFS and/or ZD7288 was applied. As shown in Figure 1(f), there were no significant differences in the PPR between all groups (on average 76%), thus indicating a negligible contamination by LPP-evoked field potentials.

### 3.2. LFS-Induced LTD in Adult Rats Is Not Affected by ZD7288

**Figure 2:**
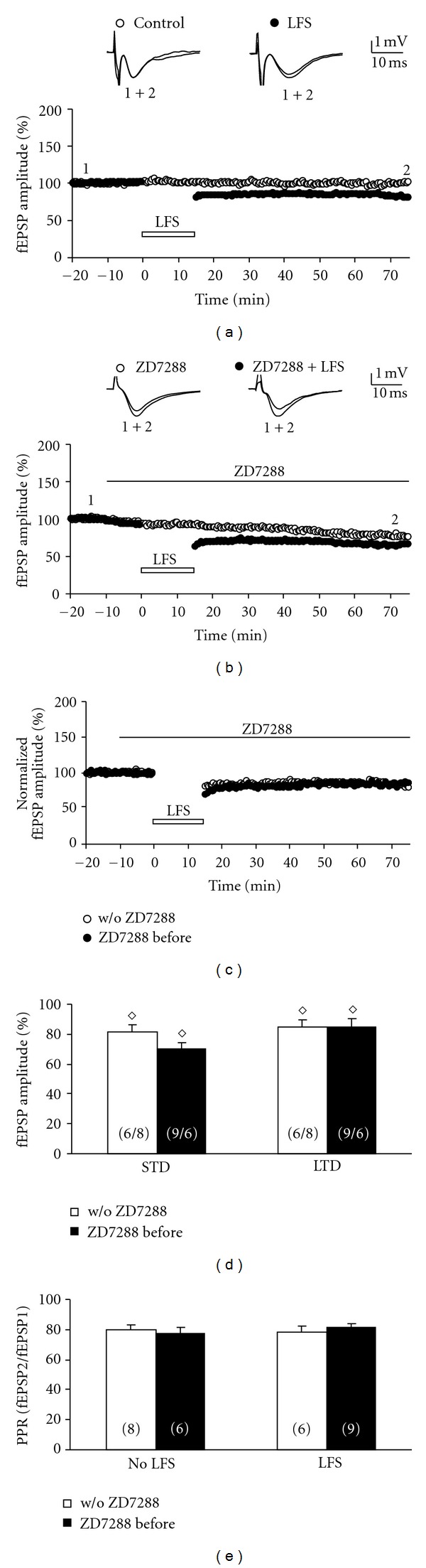
ZD7288 has no effect on LFS-induced LTD in adult rats. (a) The time course of the fEPSP amplitude following LFS in adult rats (P30–60) shows LTD (closed symbols) as compared to time-control experiments without LFS (open symbols). Representative fEPSPs were taken at the beginning (indicated by “1”) and at the end (indicated by “2”) of the experiment. (b) Comparing the fEPSP amplitude time course following ZD7288 treatment (starting 10 min prior to LFS, indicated by a closed bar) between LFS-treated (closed symbols) and non-LFS-treated time-control experiments (open symbols) reveals a synaptic depression which was similar to that in non-ZD7288 conditions shown in (a). (c) Normalized time courses of fEPSP amplitudes in both conditions: without ZD7288 (open symbols) and ZD7288 before LFS (closed symbols). Note that similar levels of LFS-induced STD and LTD were obtained in both slices without ZD7288 and slices with ZD7288 before LFS. (d) The bar graph summarizes the STD and LTD levels in both ZD7288 and non-ZD7288 conditions. The number of slices is given in parentheses (number of LFS-treated slices/number of non-LFS-treated slices). (e) The bar graph summarizes the paired-pulse ratio of all experimental groups recorded at the beginning of each experiment, that is, before ZD7288 and/or LFS were applied to the slices. Note that the PPR was similar in all groups. All data are mean ± SEM.

The results presented so far indicated a constraining effect of HCN channels on LFS-induced LTD in early postnatal MPP-dentate gyrus synapses. We then asked the question whether this LTD enhancement following ZD7288 treatment was due to an inhibition of presynaptic HCN channels that are abundant in immature MPP, but disappear with aging [4]. We therefore repeated these experiments with adult rats (P30-60). In slices taken from these animals, LFS applied to the MPP caused a short-term depression to 82 ± 2% of the baseline fEPSP amplitude (Figure 2(a), closed symbols, *n* = 6) and resulted in stable LTD of 81 ± 3% of baseline at 60 minutes after LFS. Interleaved time-control experiments without LFS demonstrated that our recording conditions were again stable for at least 90 minutes (100 ± 2% at the end of the experiment; open symbols in Figure 2(a), *n* = 8). As in early postnatal rats, we studied the effect of 10 *μ*M ZD7288 on LFS-induced LTD and thus applied this compound 10 minutes prior to LFS (Figure 2(b), closed symbols, *n* = 9). Under HCN channel blocking conditions, both STD and LTD appeared to be more depressed as compared to slices without ZD7288 treatment (63 ± 4% and 65 ± 4%, *n* = 9). However, this enhanced STD and LTD could again be biased by ZD7288-mediated synaptic depression, and interleaved time-control experiments without LFS (Figure 2(b), open symbols, *n* = 6) indeed revealed a significant synaptic depression to 77 ± 4% at the end of the experiment (open symbols in Figure 2(b)). Therefore, it was again necessary to normalize LFS-treated slices (closed symbols in Figures 2(a) and 2(b)) to non-LFS-treated slices (open symbols in Figures 2(a) and 2(b)). In contrast to slices from early postnatal rats, the normalized values for both STD and LTD were no longer significantly different between ZD7288-treated and ZD7288-untreated conditions (STD with ZD7288: 70 ± 4%, STD control: 81 ± 5%; LTD with ZD7288: 85 ± 5%, LTD control: 85 ± 6%, Figures 2(c) and 2(d)). Thus, HCN channel activation failed to have a significant effect on STD and LTD in adult rats.  Figure 2(e) illustrates again that there were no significant differences in the PPR between all groups (on average 80%), a contamination by LPP-evoked field potentials is therefore unlikely.

**Figure 3:**
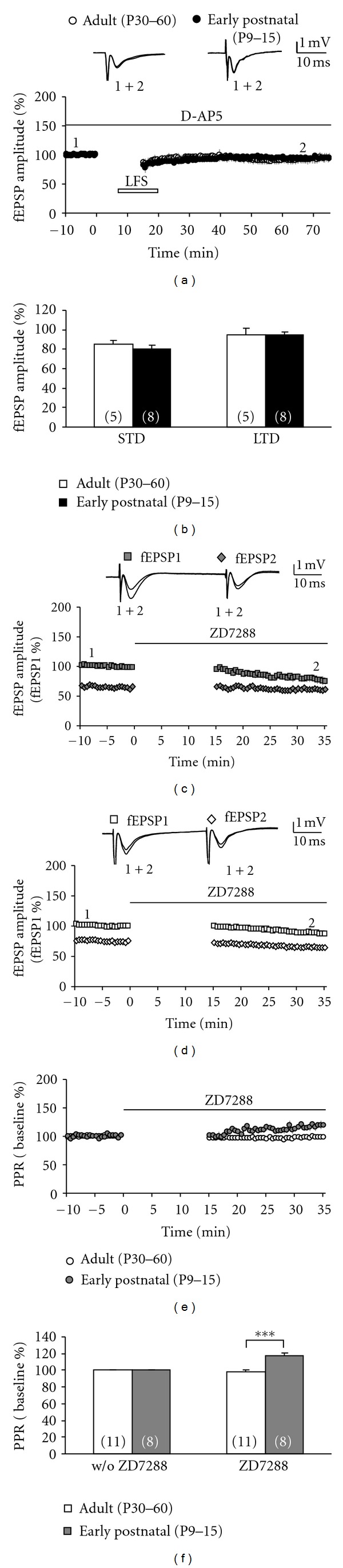
ZD7288 enhances the paired-pulse ratio in early postnatal, but not adult, rats. (a) The time course of the fEPSP amplitude following LFS in adult (P30–60, open symbols) and early postnatal rats (P9–15, closed symbols) shows no LTD in the presence of the NMDA receptor blocker D-AP5 (50 *μ*M, indicated by a black bar). Representative fEPSPs were taken at the beginning (indicated by “1”) and at the end (indicated by “2”) of the experiment. (b) The bar graph summarizes the STD and LTD levels in both adult (open bars) and early postnatal rats (closed bars) in the presence of D-AP5. (c) The time course of the fEPSP amplitudes (normalized to the fEPSP1 baseline) following paired-pulse stimulation (interpulse interval 40 ms) delivered to the MPP in early postnatal rats (P9–15) shows that ZD7288 (indicated by a black bar) depressed the first response (fEPSP1, gray boxes) more markedly than the second response (fEPSP2, gray diamonds). Representative fEPSPs were taken at the beginning (indicated by “1”) and at the end (indicated by “2”) of the experiment. (d) The time course of the fEPSP amplitudes (normalized to the fEPSP1 baseline) following paired-pulse stimulation (interpulse interval 40 ms) delivered to the MPP in adult rats (P30–60) shows that ZD7288 (black bar) depressed the first (fEPSP1, open boxes) and the second response (fEPSP2, open diamonds) to the same degree. Representative fEPSPs were taken at the beginning (indicated by “1”) and at the end (indicated by “2”) of the experiment. (e) Time courses of the PPR (normalized to baseline PPR) in both age groups: adult (open symbols) and early postnatal rats (gray symbols). Note that ZD7288 increased PPR only in early postnatal animals. (f) The bar graph summarizes the normalized paired-pulse ratio of both age groups recorded before and after ZD7288 treatment. ZD7288 significantly increased the PPR in early postnatal, but not in adult, animals. All data are mean ± SEM.

Our results may suggest that HCN channel-mediated suppression of LFS-induced LTD is downregulated during developmental maturation. However, one could argue that LFS-induced LTD in different ages may involve different induction mechanisms. We therefore asked whether NMDA receptors are required for LTD induction in both age groups and repeated our experiments in the presence of the NMDA receptor blocker D-AP5 (50 *μ*M). We in fact found that LTD was abolished under these conditions in both adult (95 ± 7%, *n* = 5) and early postnatal rats (95 ± 3%, *n* = 8; Figures 3(a) and 3(b); both age groups *P* < 0.05 versus untreated slices). Hence, differential NMDA receptor dependence of LTD induction cannot explain the differences in immature and adult tissues. However, immature MPP axon terminals express presynaptic HCN channels that have been demonstrated to be downregulated upon maturation [4]. Thus, we asked whether ZD7288 might alter the paired-pulse ratio (PPR) in slices from early postnatal and adult rats (Figures 3(c), 3(d), 3(e), and 3(f)). Initial experiments revealed that bath application of ZD7288 occasionally led to an exacerbation of disinhibitory network activity, probably due to gabazine which was present in all our solutions. Therefore, we first recorded a baseline with paired-pulse stimulation, then incubated the slices with ZD7288 (10 *μ*M) while switching off the stimulation for 15 min, and finally delivered paired-pulse stimulation again for another 20 min (Figures 3(c) and 3(d)). As shown previously (Figures 1(b) and 2(b)), ZD7288 generally depressed MPP-evoked fEPSPs. However, in slices from immature rats, the field potential evoked by the first stimulus (fEPSP1, gray boxes in Figure 3(c)) appeared to be more affected by this compound than the second response (fEPSP2, gray diamonds in Figure 3(c)). As a consequence, ZD7288 caused a significant increase of the normalized paired-pulse ratio (117 ± 4%, *n* = 8, gray symbols/bars in Figures 3(e) and 3(f)). In adult rats, however, ZD7288 depressed the first and second fEPSP to the same degree resulting in no significant change of the PPR (98 ± 2%, *n* = 11, open symbols/bars in Figures 3(e) and 3(f)). These findings indicate that HCN channel blockade causes a reduction of fEPSP amplitudes in both immature and adult tissues. But in addition, it leads to an increase of the paired-pulse ratio in immature synapses only suggesting that presynaptic HCN channels facilitate glutamate release.

### 3.3. LFS-Induced LTD in Early Postnatal, but Not in Adult, Rats Is Enhanced by L-NAME

**Figure 4:**
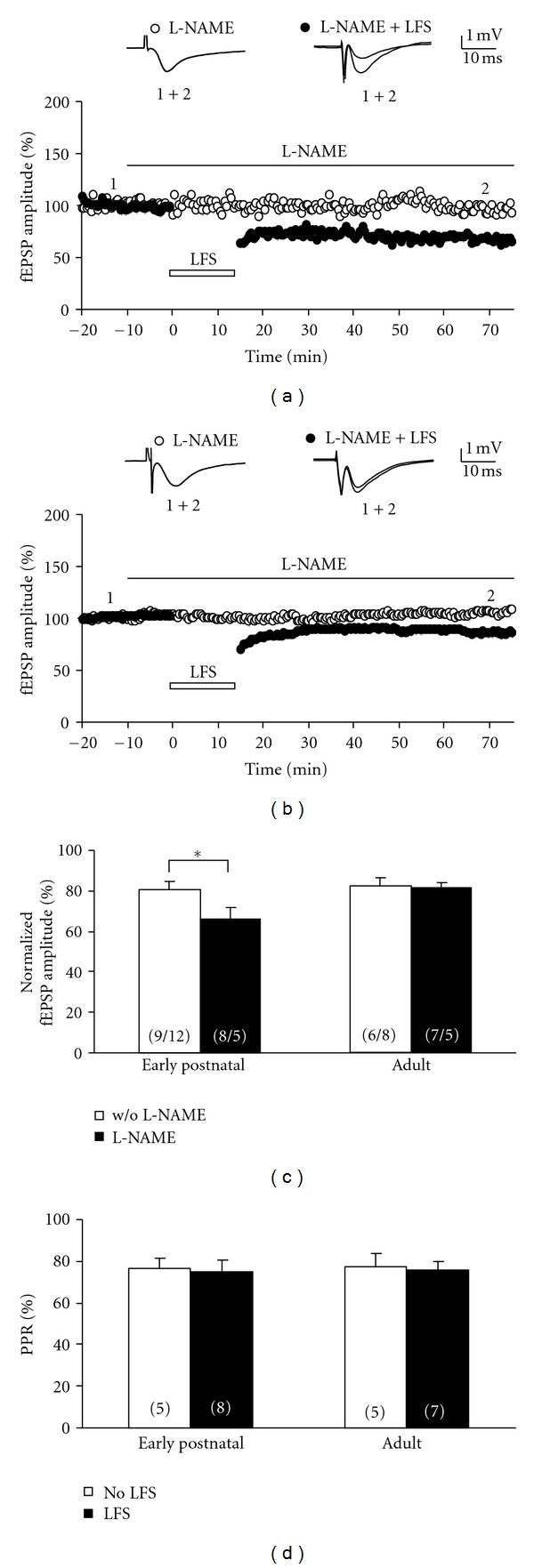
L-NAME enhances LFS-induced LTD in early postnatal, but not adult, rats. (a) The time course of the fEPSP amplitude following LFS in early postnatal rats (P9–15) shows robust LTD (closed symbols) as compared to time-control experiments without LFS (open symbols). L-NAME (100 *μ*M) was applied 10 min prior to LFS (indicated by a lack bar). Representative fEPSPs were taken at the beginning (indicated by “1”) and at the end (indicated by “2”) of the experiment. (b) The time course of the fEPSP amplitude following LFS in adult rats (P30–60) also shows LTD (closed symbols) as compared to time-control experiments without LFS (open symbols). L-NAME (100 *μ*M) was applied 10 min prior to LFS (indicated by a lack bar). Note that less LTD is obtained compared to early postnatal animals (panel A). Representative fEPSPs were taken at the beginning (indicated by “1”) and at the end (indicated by “2”) of the experiment. (c) The bar graph summarizes the LTD levels for slices treated with L-NAME (closed bars). For the sake of clarity, data from Figures 1(e) and 2(d) is also shown in order to illustrate non-L-NAME conditions (open bars). The number of slices is given in parentheses (number of LFS-treated slices/number of non-LFS-treated slices). (d) The bar graph summarizes the paired-pulse ratio of all experimental groups recorded at the beginning of each experiment, that is, before L-NAME and/or LFS were applied to the slices. Note that the PPR was similar in all groups. All data are mean ± SEM.

Our findings so far indicate that LFS-induced LTD is NMDA receptor dependent and—in the case of early postnatal synapses—is compromised by HCN channel activation. If indeed presynaptic HCN channels might be responsible for this modulation in immature tissue, a retrograde signaling pathway has to be postulated to transfer the information of postsynaptic NMDA receptor activation to presynaptic HCN channels. One major retrograde messenger is nitric oxide (NO) produced by postsynaptic NO synthase (NOS) which travels through the synaptic cleft to activate presynaptic guanylate cyclases [10, 11]. We tested this hypothesis by repeating our experiments in the presence of the NOS blocker L-NAME (100 *μ*M, Figure 4). L-NAME itself had no affect on MPP-evoked fEPSPs in both age groups (early postnatal: open symbols in Figure 4(a); adult: open symbols in Figure 4(b)). However, LFS-induced LTD was more pronounced in slices from immature rats (closed symbols in Figure 4(a)) compared to those from adult animals (closed symbols in Figure 4(b)). Normalization to non-LFS-treated slices revealed that L-NAME significantly enhanced LTD in early postnatal tissue (66 ± 6%, *n* = 7), when compared to control conditions (80 ± 4%, *n* = 9; *P* < 0.05, Figure 4(c)). This modulatory role of L-NAME was lost in adult tissue (with L-NAME: 81 ± 3%, *n* = 7; without L-NAME: 85 ± 6%, *n* = 6, Figure 4(c)). The PPR analysis of all L-NAME-treated slices (on average 78%) did not detect any significant differences, thus arguing against LPP contamination (Figure 4(d)). Our results obtained with L-NAME are reminiscent of the effect of HCN channel inhibition on LFS-induced LTD and suggest that retrograde signaling with NO might play a major role in the linkage between postsynaptic NMDA receptor activation and presynaptic HCN channel function.

## 4. Discussion

In the present study, we found that bath application of the HCN channel blocker ZD7288 depressed synaptic transmission at medial perforant path (MPP) synapses. In addition, low-frequency stimulation (LFS) delivered to these fibers resulted in LTD which was significantly enhanced after ZD7288 during the early postnatal age, but not in adult animals. Hence, HCN channel activation during neuronal activity may compromise the propensity of LTD obtained at these synapses. Unfortunately, the explanation of mechanisms mediated by ZD7288 in synaptic function may be confounded by other effects such as its action on glutamate receptors [5, 9]. While LTD was found to be NMDA receptor dependent in both age groups, HCN channels are predominantly expressed on MPP axon terminals at early postnatal ages, but this presynaptic location is lost during development [4]. Albeit postsynaptic expression of HCN channels on granule cell dendrites cannot entirely be excluded, there were no differences between slices from early postnatal and adult animals [4]. In the present study, we in fact found a significant increase of the paired-pulse ratio by HCN channel inhibition in early postnatal, but not in adult, tissue, confirming a facilitating role of presynaptic HCN channels in transmitter release. Therefore, the differential effect of ZD7288 on LFS-induced LTD in these two age groups strongly supports the idea that presynaptic HCN channels contribute to the ZD7288-mediated LTD enhancement.

How can presynaptic HCN channels modulate LTD? In general, activity-dependent changes in synaptic strength may arise from both pre- and postsynaptic mechanisms. In hippocampal dendrites, it was proposed that HCN channels have at least two major effects, that is, (i) an active shunting conductance and (ii) a tonic depolarization leading to a reduced excitatory postsynaptic potential (EPSP) amplitude and a compromised temporal summation [12–17]. On the other side, presynaptic HCN channels have been suggested to increase the reliability of neurotransmitter release via exerting a tonic depolarization on the presynaptic membrane [18]. The underlying mechanisms may involve interaction with T-type Ca^2+^ channels as shown recently in axons terminating on entorhinal layer III pyramidal cells, where the HCN channels were found to influence Ca_v_3.2 activity [19]. Reducing presynaptic HCN activity could thus result in an altered frequency [20, 21] or failure rate [22] of action potential-dependent transmitter release.

Importantly, HCN channels are very sensitive to changes of the intracellular milieu, such as altered levels of PIP2 or cyclic AMP [23–25]. Interestingly, cyclic GMP may also stimulate HCN channel function [11], and activation of guanylyl cyclases is the typical downstream effect of the NO retrograde signaling pathway [26]. There is indeed a large body of evidence supporting the role of NO in modulating synaptic transmission in the CA1 region of the hippocampus [10, 27], reviewed in [26], but the role of NO in the molecular layer of the dentate gyrus has attracted less attention. One study found that NO synthase inhibition blocked both LTP and LTD at these synapses [28]. At least in our hands, however, L-NAME had no effect on medial perforant path-evoked LTD in slices from adult rats, and in immature tissue, we found a significant increase of LTD values. Albeit the reasons for this discrepancy remain unclear, the results of the present study suggest that postsynaptic NMDA receptor activation leads to NO synthesis acting as a retrograde messenger traveling from the postsynaptic site to the presynaptic terminal. Within the presynapse, we propose the activation of NO-dependent guanylyl cyclases and thereby production of cGMP which facilitates HCN channel function [11]. Both NO production and HCN channel activation seem to compromise LTD at immature synapses, since inhibition of these pathways enhanced LTD.
